# Syringin (Sinapyl Alcohol 4-*O*-Glucoside) Improves the Wound Healing Capacity of Fibroblasts and Keratinocytes In Vitro

**DOI:** 10.3390/ijms26167827

**Published:** 2025-08-13

**Authors:** Andrzej Parzonko, Agnieszka Filipek, Marcin Równicki, Anna K. Kiss

**Affiliations:** 1 Chair and Department of Pharmaceutical Biology, Medical University of Warsaw, Banacha 1, 02-097 Warsaw, Poland; agnieszka.filipek@wum.edu.pl; 2 Microbiota Lab, Department of Pharmaceutical Microbiology and Bioanalysis, Medical University of Warsaw, Banacha 1, 02-097 Warsaw, Poland; marcin.rownicki@wum.edu.pl

**Keywords:** syringin, wound healing, skin fibroblasts, keratinocytes, migration, Smad2/Smad3 signaling pathway, COL1A1, ACTA2

## Abstract

Wound healing is a complex process in which TGFβ plays a key role. Previous studies have shown that syringin, a phenylpropanoid glycoside present in lilac bark (*Syringa vulgaris* L.), stimulates TGFβ expression in human monocyte-derived macrophages in addition to inhibiting the secretion of pro-inflammatory cytokines. Here, we investigated the effect of syringin on migration, invasion, and TGFβ production, as well as the effect on the release of pro-inflammatory cytokines in human dermal fibroblasts (NHDF) and keratinocytes (HaCaT) and its mechanism of action. NHDF and HaCaT cells were treated with the tested compound (12.5–100 µM), and a scratch assay was performed. The effect of migration using modified Boyden chambers was analyzed. TGFβ and IL-6 release were also assessed using ELISA kits. Cell proliferation was assessed using MTT and BrdU incorporation tests, while cytotoxicity was assessed using a neutral red uptake test. Smad2 and Smad3 phosphorylation were assessed using Western Blotting. ACTA2, COL1A1, and TIMP3 expression was analyzed using qPCR. Cells treated with syringin showed an increase in invasion potential in the scratch assay. A significant increase in skin fibroblast migration through the porous membrane was also observed. Syringin increased TGFβ release and inhibited IL-6 release by NHDF and HaCaT cells. No effect of syringin on cell proliferation or cytotoxic effects was observed. Western blot analysis showed significant activation of Smad2 and Smad3 in the presence of syringin in NHDF cells, but not in HaCaT. Quantitative PCR analysis revealed a strong increase in ACTA2 and COL1A1 gene expression in fibroblast cells treated with syringin. The present study demonstrated that syringin present in *S. vulgaris* stem bark increased dermal fibroblasts and keratinocytes’ wound healing function through activation of cell migration.

## 1. Introduction

Human skin accounts for 15–20% of body weight, making it the largest organ of the human body. It is the first line of defense against environmental hazards as a physical barrier and maintains the balance between the body and the environment. It protects the body from mechanical and chemical agents, UV radiation, trauma, and microbial penetration. It also enables thermoregulation and maintenance of water and electrolyte homeostasis. Restoring its continuity after injury is particularly important for the functioning of the body [1].

Wound healing in the skin is a dynamic and highly regulated process of cellular, humoral, and molecular mechanisms, which starts immediately after an injury and can last for years [2]. Wound healing involves complex chemical reactions of biologically active and locally active substances and physical phenomena such as an increase in tensile strength and changes in skin elasticity [3]. The wound healing process consists of three phases: the inflammatory phase, the proliferative phase, and the maturation and remodeling phase [4]. A prolonged inflammatory process at the site of skin damage hinders the healing process due to the delay and obstruction of the restoration of tissue continuity [5]. An important factor accelerating the regeneration process of damaged skin is the TGFβ factor, which stimulates skin cell migration and proliferation, accelerating the restoration of tissue continuity at the site of injury [6,7,8]. The subject of this research is the search for substances of natural origin that have a beneficial effect on the wound healing process by stimulating the secretion of TGFβ by cells while inhibiting chronic inflammation.

**Figure 1:**
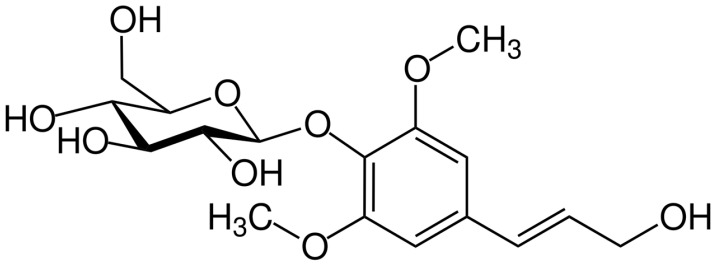
Chemical structure of syringin.

Syringin (sinapyl alcohol 4-*O*-glucoside, Figure 1) is a phenylpropanoid glucoside first isolated from the bark of lilac (*Syringa vulgaris* L., Oleaceae) [9]. It has since been found to be distributed throughout selected types of plants used in traditional medicine as food (e.g., *Tinospora crispa* (L.) Hook.f. & Thomson, *Codonopsis pilosula* (Franch.) Nannf., and *Cirsium setidens* (Dunn) Nakai). Due to the fact that it is one of the main constituents of *Eleutherococcus senticosus* (Rupr. & Maxim.) Maxim. (Araliaceae), “Siberian ginseng”, it is also known as eleuteroside B. It has been documented that syringin shows multiple pharmacological properties. It has been shown to have antidepressant [10], anticancer, anti-inflammatory, antioxidant [11,12,13,14], cardioprotective [15], and neuroprotective properties [16,17,18]. It may have a protective effect on the kidneys and has the ability to reduce inflammation and oxidative stress [19]. Studies have shown that syringin can reduce the expression of pro-inflammatory cytokines in some diseases. The results suggest that syringin exhibits a protective effect on intestinal cells by inhibiting NF-κB, while activating the Nrf2 signaling pathway in colitis [20]. One study confirmed that syringin inhibits the proliferation and migration of breast cancer cells and promotes their apoptosis [21].

Previously presented in vitro studies have been carried out showing that syringin, the main component of lilac bark extract, has the ability to stimulate monocytes/macrophages to secrete TGFβ [22]. Both bark extract and syringin have also been shown to inhibit the secretion of pro-inflammatory cytokines, such as IL-8 or TNFα, and reactive oxygen species. The role of TGFβ in wound healing is crucial, while prolonged inflammation at the wound site slows and prolongs the healing process. Based on previous research, it seemed important to investigate the potential effects of syringin on the processes leading to wound healing in a model of human fibroblasts and keratinocytes. The aim of the study was to investigate whether syringin, the main component of some medicinal plants, increased dermal fibroblasts and keratinocytes wound healing function through activation of cell migration, which would enable the use of their healing-accelerating and anti-inflammatory properties in the treatment of hard-to-heal wounds and ulcers of the skin and mucous membranes, and to elucidate the molecular mechanism of its action.

## 2. Results

### 2.1. Syringin Promotes Fibroblasts and Keratinocytes Migration in Scratch Assay

**Figure 2:**
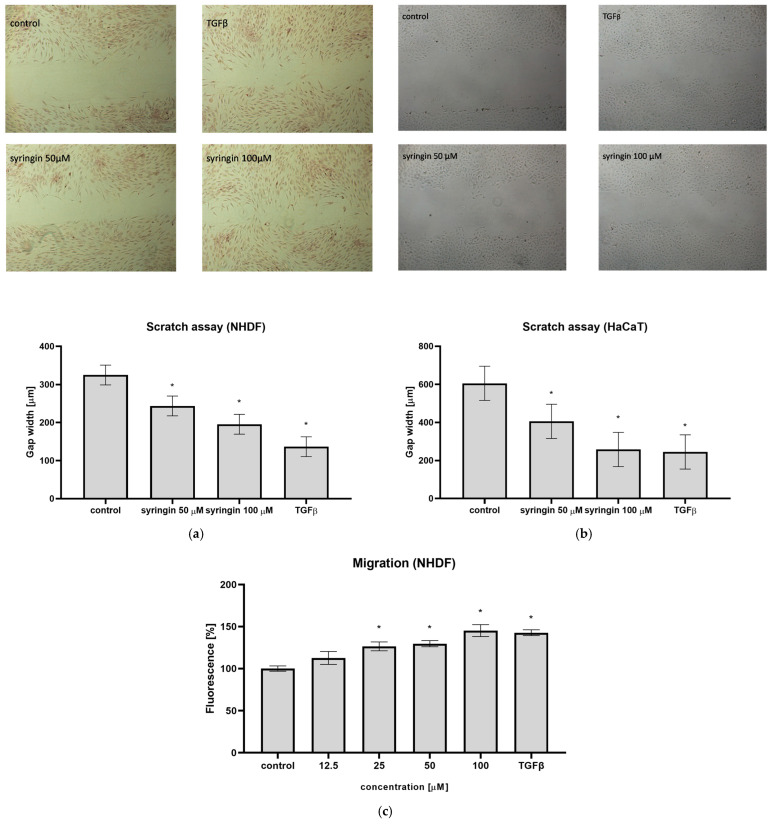
Syringin increased cell invasion and migration. (**a**) Stimulating effect on NHDF cell invasion in scratch assay. Representative photomicrographs of the cells (upper) and impact on gap width (lower). The results are the mean ± SD of three independent assays performed in triplicate (*n* = 3); (*)—the significant differences (*p* < 0.05) relative to the untreated cells. (**b**) Stimulating effect on HaCaT cells’ invasion in scratch assay. Representative photomicrographs of the cells (upper) and impact on gap width (lower). The results are the mean ± SD of three independent assays performed in triplicate; (*)—significant differences (*p* < 0.05) relative to the untreated cells. (**c**) Stimulating effect on NHDF cell migration through the porous membrane; the results are the mean ± SD of three independent assays performed in triplicate (*n* = 3); (*)—significant differences (*p* < 0.05) relative to the untreated cells.

The ability of syringin to stimulate fibroblast and keratinocyte migration was detected by a scratch assay. As shown in Figure 2a, incubation of the cells in the presence of syringin at concentrations of 50 and 100 µM for 24 h resulted in increased cell migration to the scratch site. The measured gap width was significantly smaller in the presence of syringin compared to control cells. In the case of immortalized keratinocytes, the observed effect was similar; incubation of the cells in the presence of syringin resulted in an increased number of cells migrating to the site of the scratch (Figure 2b). For a syringin concentration of 100 µM, the observed effect was comparable to TGFβ at a concentration of 10 ng/mL, which was used as a positive control.

### 2.2. Syringin Promotes Fibroblasts Migration Through Porous Membrane

The ability of syringin to stimulate fibroblasts’ migration was also assessed using the Boyden chamber assay. Syringin in a concentration range of 12.5–100 µM stimulated cell migration through a porous membrane in a concentration-dependent manner, achieving a 45.2% increase compared to the control for a concentration of 100 µM. This effect was comparable to TGFβ at a concentration of 10 ng/mL, for which a 42.7% increase in migration was observed (Figure 2c).

### 2.3. Syringin Does Not Affect Fibroblast or Keratinocyte Proliferation

**Figure 3:**
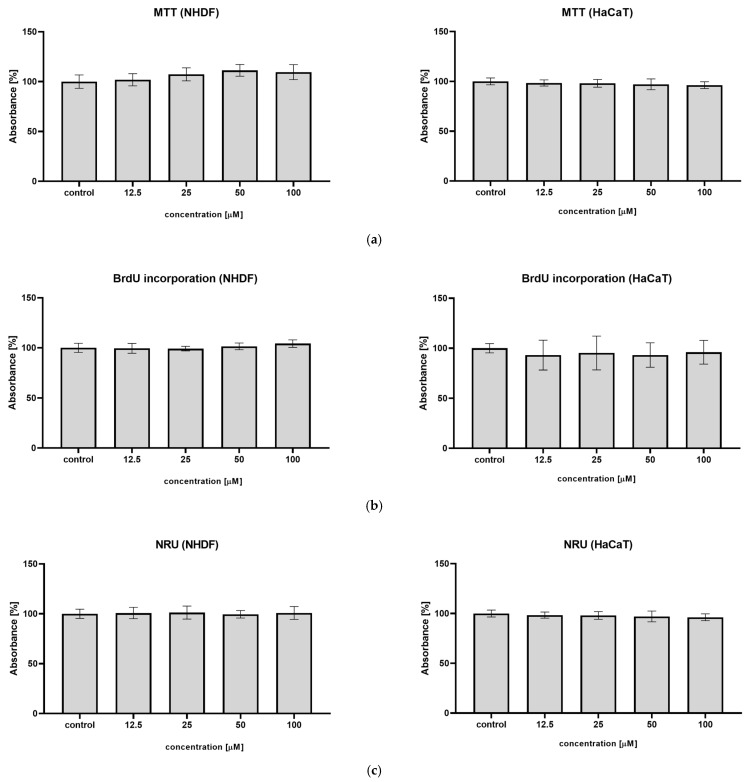
Syringin has no effect on NHDF and HaCaT cell proliferation. (**a**) Proliferative activity determined by the MTT assay. (**b**) Proliferative activity determined by the BrdU incorporation assay. (**c**) Cytotoxicity determined by the NRU assay. The cell viability was calculated as absorbance ratio compared to untreated cells. The results are the mean ± SD of three independent assays performed in triplicate (*n* = 3).

An MTT assay was performed to evaluate the influence of syringin on the metabolic activity of cells. In the presence of syringin, cell viability for all concentrations tested was similar to the controls, represented by untreated cells, after 24 h (Figure 3a). For fibroblasts, a slight increase was observed at a concentration of 50 µM; however, this was without statistical significance.

The BrdU assay was performed to analyze cell proliferation. The test revealed that treatment with syringin in a concentration range of 12.5–100 µM does not affect the proliferation of the cell populations analyzed (Figure 3b). For keratinocytes, a slight decrease was observed at all concentrations tested; however, this was without statistical significance.

The NRU assay was performed to determine the cytotoxic effects of syringin on cells. No differences between syringin-treated cells across the concentration range and controls indicate that syringin has no cytotoxic effect on fibroblasts and keratinocytes (Figure 3c).

### 2.4. Syringin Stimulates TGFβ Release by Fibroblasts and Keratinocytes

**Figure 4:**
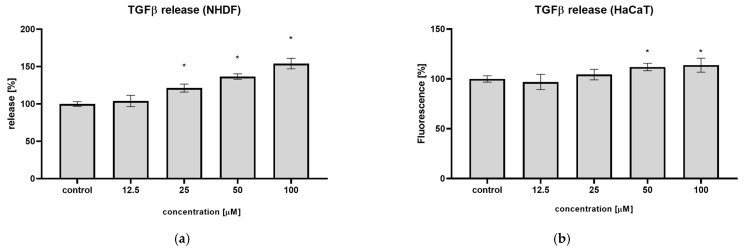
Syringin stimulates TGFβ secretion by NHDF cells (**a**) and HaCaT cells (**b**). The results are the mean ± SD of three independent assays performed in triplicate (*n* = 3); (*) indicates significant differences (*p* < 0.05) relative to the untreated cells.

An ELISA test was performed to evaluate the effect of syringin on TGFβ secretion by cells. Syringin increased TGFβ secretion by NHDF cells by 21.2, 36.6, and 54.0% at concentrations of 25, 50, and 100 µM, respectively (Figure 4a). A weaker effect was observed on keratinocytes; here, the increase in TGFβ secretion was 4.4, 11.9, and 13.8%, respectively, at the same concentrations (Figure 4b).

### 2.5. Syringin Inhibits IL-6 Secretion by Fibroblasts and Keratinocytes

**Figure 5:**
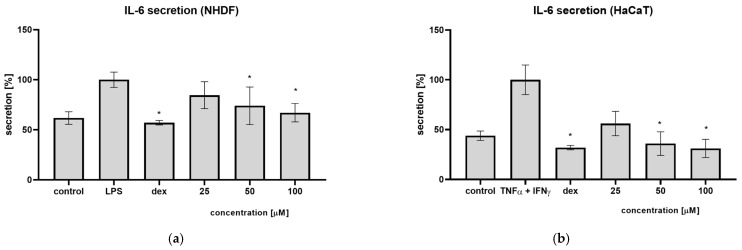
Syringin inhibits IL-6 secretion. (**a**) NHDF cells were stimulated with LPS. The results are the mean ± SD of three independent assays performed in triplicate; (*) indicates significant differences (*p* < 0.05) relative to the LPS-treated cells. (**b**) HaCaT cells were stimulated by TNFα/IFNγ. The results are the mean ± SD of three independent assays performed in triplicate (*n* = 3); (*) indicates significant differences (*p* < 0.05) relative to the TNFα/IFNγ-treated cells.

An ELISA test was performed to evaluate the effect of syringin on IL-6 secretion. Pre-incubation of fibroblasts in the presence of syringin reduces the amount of secreted interleukin 6 after stimulation with LPS by 15.5%, 26.1%, and 33.0% at concentrations of 25, 50, and 100 µM, respectively (Figure 5a). In a test on keratinocytes stimulated with TNFα and interferon-γ, in the presence of syringin, a decrease in IL-6 secretion by 14.2%, 34.4%, and 41.7% at concentrations of 25, 50, and 100 µM, respectively, was observed.

### 2.6. Syringin Increases Smad2 and Smad3 Phosphorylation in Fibroblasts

**Figure 6:**
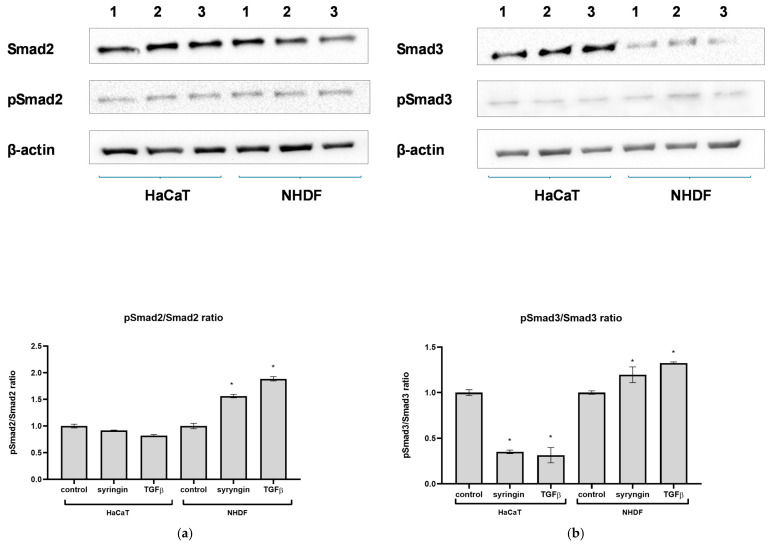
Syringin modulates Smad2 and Smad3 expression and phosphorylation. (**a**) Relative Smad2 and phospho-Smad2 protein expression in HaCaT and NHDF cells after stimulation with syringin (100 µM); 1–3 represent the control cells, syringin-stimulated cells, and TGFβ-stimulated cells. (**b**) Relative Smad3 and phospho-Smad3 protein expression in HaCaT and NHDF cells after stimulation with syringin (100 µM); 1–3 represent the control cells, syringin-stimulated cells, and TGFβ-stimulated cells. The results are the mean ± SD of three independent assays (*n* = 3). (*) indicates significant differences (*p* < 0.05) relative to the untreated cells.

To clarify whether the mechanism of action of syringin is related to the activation of the Smad2/Smad3 pathway, Western blot analysis was performed. In the presence of syringin at a concentration of 100 µM, there was a small but significant decrease in the level of Smad2 in fibroblast cells, with a concomitant increase in the level of phosphorylated Smad2 (pSmad2). At the same time, a slight increase in Smad3 levels (by 65.8%) and a significant (by 96.8%) increase in pSmad3 levels in fibroblast cells were observed (Figure 6). Consequently, a significant increase in phosphorylated Smad2 (pSmad2/Smad2 ratio) and phosphorylated Smad3 (the pSmad3/Smad3 ratio) was observed in syringin-treated fibroblasts. In the case of keratinocytes, the opposite effect was observed: a slight but statistically significant increase in the level of Smad2 was observed (by 18.4%), while no significant increase in the level of phosphorylated Smad2 was observed. A very large increase in the level of Smad3 was also observed, but there were no significant differences in the level of phosphorylated Smad3 compared to the control. As a result, there was a decrease in the ratio of phosphorylated Smad2 (the pSmad2/Smad2 ratio) and phosphorylated Smad3 (the pSmad3/Smad3 ratio) in syringin-treated keratinocytes compared to the control.

### 2.7. Syringin Stimulates ACTA2 and COL1A1 Expression in Fibroblasts

**Figure 7:**
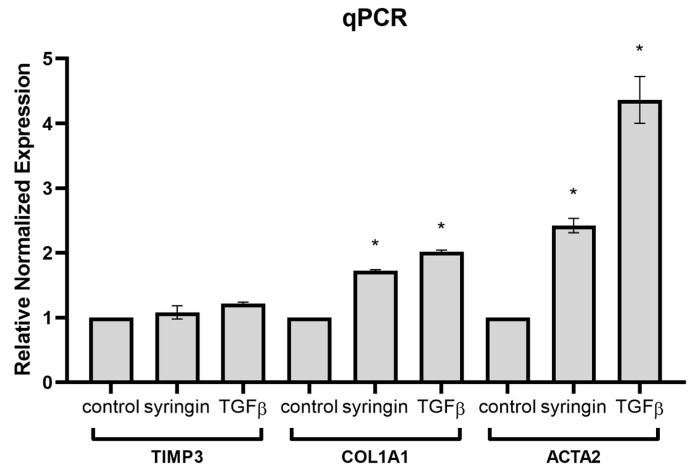
Syringin stimulates gene expression: relative normalized expression of TIMP3, COL1A1, and ACTA2 in NHDF cells after stimulation with syringin (100 µM), or TGFβ (10 ng/mL—positive control). The results are the mean ± SD of three independent assays (*n* = 3). (*) indicates significant differences (*p* < 0.05) relative to the untreated cells.

To confirm the mechanism of action of syringin on fibroblasts, the effect on the expression of genes dependent on the Smad2/3 pathway was investigated. Quantitative real-time PCR (qPCR) analysis was performed to evaluate the expression levels of TIMP3 (tissue inhibitor of metalloproteinases 3), COL1A1 (alpha 1 chain of type I collagen), and ACTA2 (smooth muscle α actin) in the presence of syringin (100 µM) and TGFβ (10 ng/mL) as a positive control, relative to the untreated cells, using the ΔΔCq method with β-actin and GAPDH (glyceraldehyde-3-phosphate dehydrogenase) as reference genes. TIMP3 expression remained unchanged in the presence of syringin, showing a non-significant 1.08-fold increase. Moderate but statistically non-significant upregulation was observed in the presence of TGFβ, with a 1.22-fold increase compared to the control. In contrast, COL1A1 expression was significantly upregulated in the presence of syringin. Cells treated with syringin exhibited a 1.73-fold increase, while cells treated with TGFβ (positive control) had a stronger 2.02-fold increase (Figure 7). The most substantial changes were observed in ACTA2 expression. Cells treated with syringin demonstrated a significant 2.42-fold upregulation, whereas cells treated with TGFβ exhibited a robust 4.36-fold increase, reflecting a strong induction in response to the experimental conditions.

## 3. Discussion

Syringin, a phenylpropanoid glycoside, is the subject of much research due to its unique sinapyl alcohol glycoside structure. Numerous in vitro and in vivo studies have demonstrated its diverse pharmacological effects, such as immunomodulatory, tumor suppression, hypoglycemic, neuroprotective, cardioprotective, hepatoprotective, and other effects [23]. Although it is included in plants with wound-healing properties [24,25], there is a lack of research on the effects of syringin on the skin and the wound healing process. In this study, human dermal fibroblasts (NHDF) and immortalized human keratinocytes (HaCaT) were used to investigate the ability of syringin to increase wound healing ability and reduce inflammation at the site of injury and elucidate the molecular mechanisms involved.

The reconstruction of damaged skin is a complex process and requires the participation of different cell types. Of particular importance for the wound healing process are fibroblasts, which migrate to the wound site, produce extracellular matrix proteins (especially collagen fibers), and, through the secretion of cytokines and growth factors, stimulate the migration and proliferation of further cells. The ability to stimulate the migration of skin cells to the wound site is the first manifestation of the substances tested to accelerate wound healing. For this reason, the ability of syringin to stimulate cell migration in a scratch assay was tested. Syringin at concentrations of 12.5–100 µM stimulated the migration of human skin fibroblasts, significantly reducing the width of the scratch on the plate (Figure 2a). A similar effect for immortalized keratinocytes was obtained; syringin also stimulated the migration of these cells in the concentration range indicated (Figure 2b). For a syringin concentration of 100 µM, the observed effect was comparable to TGFβ at a concentration of 10 ng/mL for both cell lines. In addition, in a cell migration assay across a porous membrane, an increase in migration of NHDF fibroblasts in the presence of syringin was also observed, and the observed effect was concentration-dependent (Figure 2c).

The skin cells involved in wound healing, both fibroblasts and keratinocytes, proliferate intensively at the wound site to rebuild the damaged tissue. Excessive cell proliferation can lead to hypertrophic changes and scar formation. The effect of syringin on cell proliferation in vitro was investigated. In both the MTT assay and the BrdU incorporation assay, it was shown that syringin did not affect cell proliferation in the concentration range tested (Figure 3). Furthermore, in the neutral red uptake assay, we found no cytotoxic effect of syringin on fibroblasts and immortalized keratinocytes.

The results obtained in the present study are very promising, although they are different from previously published studies where syringin inhibited the proliferation and migration of breast cancer cells [21]. On the other hand, the results we present concern normal skin cells and the stimulation of their repair functions, in which different molecular mechanisms are involved than in tumor cell invasion.

The TGFβ factor plays a key role in all phases of wound healing by regulating the functions of keratinocytes, fibroblasts, endothelial cells, monocytes, and other cell types [5,6]. It is responsible for initiating inflammation at the site of injury and for the formation of granulation tissue, inducing alpha-actin expression in fibroblasts and the differentiation process of myofibroblasts, and stimulating angiogenesis by upregulating the VEGF factor. It is also a key factor in promoting skin cell migration, including keratinocytes, during wound closure and re-epithelialization. Previous results on neutrophils and monocytes have shown that syringin stimulates these cells to secrete TGFβ [22]. In the present study, a similar effect of syringin toward human fibroblasts was observed; syringin increased TGFβ secretion by NHDF up to 54.0% at the concentration of 100 µM, respectively (Figure 4). A weaker effect was observed on keratinocytes, where the highest increase in TGFβ secretion was 13.8% at the same concentration. The observed effect may be beneficial to the wound healing process, as cells stimulated by syringin to secrete TGFβ may stimulate further cells to migrate to the wound site, accelerating wound closure.

Often, the cause of prolonged wound healing is a prolonged inflammatory phase, which does not allow the wound to progress to the proliferative phase. For this reason, we investigated the effect of syringin on the secretion of pro-inflammatory cytokines by skin cells. Previous studies have shown that syringin has the ability to inhibit IL-6 secretion by monocyte-macrophages and neutrophils. In the present study, the ability of syringin to inhibit IL-6 secretion from fibroblasts and keratinocytes (the main cells involved in the wound healing process) was tested. It was observed that pre-incubation of fibroblasts in the presence of syringin reduces the amount of secreted interleukin 6 after stimulation with LPS (Figure 5). Syringin showed a similar effect against keratinocytes stimulated with TNFα and interferon-γ. In the presence of syringin, a decrease in IL-6 secretion was observed. While the secretion of pro-inflammatory cytokines like interleukins is essential in the inflammatory phase of the healing process, they can disrupt this process in the proliferative phase. For this reason, syringin’s inhibition of the secretion of pro-inflammatory cytokines may facilitate the transition from the inflammatory phase of the process to the proliferative phase, accelerating the healing process.

Having demonstrated the stimulatory effect of syringin on skin cell migration, the next step was to try to elucidate the possible molecular mechanism of this action. As syringin was not shown to stimulate cell proliferation (skin fibroblasts or keratinocytes) but rather only their migration, the next step focused on the TGFβ signaling pathway as a possible site of action for syringin. The major intracellular mediators of TGFβ signaling are Smad proteins, and Smad2 and Smad3 play an especially important role in cell migration [6,8]. Activated TGFβRII binds and phosphorylates receptor-activated Smad2 or Smad3, which, upon heterodimerization with Smad4, translocate into the nucleus. Within the nucleus, activated Smad complexes become transcriptional factors. Western blotting analysis showed that in the presence of syringin, there is an increase in the expression of Smad3 but not Smad2 in NHDF fibroblasts (Figure 6). At the same time, a very large increase in the level of phosphorylated Smad3 was observed in fibroblasts in the presence of syringin, and we observed a slightly smaller increase in the level of phosphorylated Smad2 in these cells. While syringin significantly increased the level of Smad3 in fibroblast cells, it increased the level of phosphorylated Smad3 (pSmad3) much more strongly, suggesting that this mechanism (Smad2/Smad3 activation) may be responsible for the migration-stimulating effects of syringin on fibroblast cells. Quite different results were obtained for immortalized keratinocytes (HaCaT). In the presence of syringin, a large increase in Smad3 levels and a weaker increase in Smad2 levels in these cells were observed. However, in contrast to the fibroblast assay, syringin only slightly increased phosphorylated Smad2 levels, while it caused a slight decrease in phosphorylated Smad3 levels, but these differences were not statistically significant. Smads are critical for TGFβ signaling; however, some studies have suggested that Smad-independent pathways can also mediate TGFβ signal transduction. Thus, the effects of TGFβ signaling during wound healing can be achieved through both Smad-dependent and -independent signaling. In light of the above results, it can be speculated that, in the case of fibroblasts, the beneficial effect of syringin may be at least partly related to activation of the Smad2/Smad3 pathway. In contrast, in the case of immortalized keratinocytes, the results obtained do not allow a link to be established between the stimulating effect of syringin on keratinocyte migration in vitro and the activation of Smad2 and Smad3, and this mechanism needs further clarification. The results obtained for immortalized keratinocytes are somewhat surprising, as it would be expected that the level of phosphorylated Smad3 would increase, especially in the case of the positive control (TGFβ-treated cells). However, it should be considered that the HaCaT line is an immortalized cell line that, under certain conditions, may show characteristics of a tumor cell line. For this reason, these results should be approached with some caution. Nevertheless, as skin fibroblasts play a key role in the wound healing process, the beneficial effect of syringin on fibroblast function in terms of the wound healing process observed in this study looks very promising. Therefore, further studies should focus on the mechanism of action of syringin on skin fibroblasts.

Having demonstrated the stimulatory effect of syringin on Smad2 and Smad3 activation in fibroblasts, it was necessary to investigate whether syringin also stimulates the expression of Smad2/3 pathway-dependent genes. Quantitative qPCR analysis showed that in the presence of syringin, there is a significant increase in the expression of COL1A1 and ACTA2 genes in skin fibroblast cells (Figure 7). In response to TGF-β signaling in the wound environment, fibroblasts migrate, proliferate, and transform into myofibroblasts, intensively increasing the expression of ECM (especially collagen) and contractile proteins (such as α-SMA). COL1A1-encoding type 1 collagen plays a key role in the restoration of skin continuity during the wound healing process [26]. ACTA2-encoding α-SMA, on the other hand, is a marker for the transformation of fibroblasts into myofibroblasts, which occurs during the proliferative and remodeling phases of the healing process [27]. Expression of both these genes is closely linked to activation of the TGFβ pathway and is mediated by activation (phosphorylation) of Smad2/Smad3/Smad4. At the same time, no significant effect of syringin was observed on the expression of TIMP3, whose expression is also stimulated by TGFβ, but mainly via ERK1/2-activated Sp1 binding to its regulatory element in the TIMP3 promoter [28]. The results clearly indicate that syringin stimulates the expression of TGFβ-dependent genes involved in wound healing through activation of the Smad2/Smad3 pathway.

In summary, the results of the study presented suggest that syringin, present in lilac bark or eleutherococcus root, among others, has the ability to stimulate mechanisms for wound healing. This effect is related to stimulation of cell migration rather than stimulation of cell proliferation, which prevents the risk of hypertrophic changes in scar tissue. At the same time, the anti-inflammatory properties of syringin may facilitate the transition from the inflammatory to the proliferative phase of the wound healing process. Studies carried out cannot unambiguously confirm the mechanism of this action but suggest that the Smad2/Smad3 pathway is activated in normal skin fibroblasts, resulting in the stimulation of the expression of TGFβ-dependent genes important in the wound healing process, such as COL1A1 and ACTA2. The results provide a solid basis for planning further studies to justify the potential use of syringin as a wound healing accelerator.

## 4. Materials and Methods

### 4.1. Chemicals and Reagents

Syringin (4-(3-hydroxyprop-1-enyl)-2,6-dimethoxyphenyl-β-D-glucopyranoside) was isolated from *Syringa vulgaris* stem bark as previously described [21]. The purity of these compounds >95% was determined by UHPLC-DAD-MS/MS and NMR. DMEM (Dulbecco Modified Eagle Medium) medium, FBS (fetal bovine serum), PBS (phosphate-buffered saline), and cell culture reagents were purchased from Thermo Fisher Scientific Inc. (San Francisco, CA, USA). Recombinant human TGFβ1 protein (Active) was purchased from Abcam (Cambridge, MA, USA). Recombinant Human TNF-α Protein was obtained from BioTechne (R&D Systems, Minneapolis, MN, USA), Recombinant Human IFN-γ was obtained from Gibco (Invitrogen, Grand Island, NY, USA), and Dexamethasone was purchased from Sigma-Aldrich (St. Louis, MO, USA). Lipopolysaccharide from E. Coli O111:B4 was purchased from Sigma-Aldrich (St. Louis, MO, USA). Thiazolyl blue tetrazolium bromide (MTT) was purchased from Abcam (Cambridge, MA, USA). The absorbance and fluorescence were measured using a BioTek Synergy 4 microplate reader with Gen5 (BioTek Instruments, Inc., Winooski, VT, USA).

### 4.2. Cell Culture and Treatments

NHDF normal human skin fibroblasts were purchased from Lonza (Verviers, Belgium). HaCaT immortalized human keratinocytes were purchased from CLS (Eppelheim, Germany). Cells were cultivated in DMEM medium supplemented with 10% FBS and penicillin-streptomycin in a humidified incubator (37 °C, 5% CO_2_). For each experiment, syringin was dissolved in PBS, filtered, and diluted with culture medium to obtain the final concentration. LPS and TGFβ were dissolved in sterile PBS; dexamethasone was dissolved in DMSO and added to the cell culture. The DMSO concentration was below 0.5% and did not affect cell viability.

### 4.3. Scratch Assay

The scratch assay was performed using Cell Comb™ for Scratch Assay plates (Millipore, Burlington, MA, USA) according to the manufacturer’s protocol. Cells were plated on rectangular cell culture plates and cultivated for 24 h to obtain a confluent monolayer. Then, the scratches were created using the provided comb. The scratched monolayer was washed with the medium to remove detached cells, and fresh serum-free medium was added to the cells. Cells were treated with several concentrations (12.5, 25.0, 50.0, and 100 µM) of syringin or TGFβ (10 ng/mL) (positive control) and plates were incubated for another 24 h. Plates were observed under the inverted microscope Nikon TS100F equipped with a digital camera; they were then photographed, and cell invasion was assessed through gap width measurement. The width of the gap was measured at 10 points in each photomicrograph using NIS-Elements BR 3.2 software. The results were presented as the gap width (µm) in comparison to the negative control (untreated cells).

### 4.4. Cell Migration Assay

To assess the effect of syringin on skin fibroblast migration, the Cultrex^®^ Cell Migration Assay (R&D Systems, Minneapolis, MN, USA) was used according to the manufacturer’s protocol. Briefly, cells were starved by incubating them for 24 h in a serum-free medium prior to the assay. Then, cells were harvested and seeded into the top chambers of 96-well plates, whereas the cell culture medium in the presence or absence of syringin was added to the bottom chambers. TGFβ (10 ng/mL) was used as a positive control. The chambers were assembled and incubated for 24 h. After incubation, the cells were dissociated from chambers, stained with calcein AM, and their fluorescence was measured using a microplate reader at 485 nm excitation and 520 nm emission.

### 4.5. Cell Proliferation and Cytotoxicity Assay

The effect of syringin on cell proliferation was measured using the MTT and BrdU assay. For the MTT proliferation assay, cells were seeded in 96-well plates and incubated with various concentrations (12.5, 25.0, 50.0, and 100 µM) of syringin for 24 h. The culture medium was then removed, the MTT solution was added to the cells, and they were incubated for 3 h. The formed formazan crystals were dissolved in DMSO, and the absorbance was measured using a microplate reader at a wavelength of 490 nm. The BrdU Cell Proliferation ELISA Kit (colorimetric) (Abcam, Cambridge, MA, USA) was used to perform the BrdU incorporation assay. For the assay, cells were seeded in 96-well plates and then incubated in the presence of different concentrations of syringin (12.5, 25.0, 50.0, and 100 µM) and BrdU for 24 h. The cells were then fixed, and the amount of incorporated BrdU was determined by ELISA using anti-BrdU antibodies according to the manufacturer’s instructions.

Syringin’s cytotoxicity toward cells was assessed using the NRU assay. Cells were seeded in 96-well plates and incubated in the presence of different concentrations of syringin for 24 h. The medium was then removed, and a neutral red solution was added to the cells and incubated for 3 h, after which the solution was removed, the cells were washed with PBS, the dye accumulated inside the cells was dissolved in a water–ethanol–acetic acid mixture, and absorbance was measured using a microplate reader at 540 nm.

### 4.6. Cytokines Release Assay

For the assay of TGFβ secretion, cells were incubated for 24 h with syringin in various concentrations, and then supernatants were collected. The quantity of TGFβ was measured using the BD OptEIA human TGF beta ELISA Kit (BD Biosciences, Franklin Lakes, NJ, USA), according to the manufacturer’s protocols. To assess the effect of syringin on the release of IL-6, cells were seeded in 12-well plates, preincubated in the presence of different concentrations of syringin or 5 µM dexamethasone (positive control) for 1 h, and stimulated with 100 ng/mL LPS (NHDF) or 10 µM interferon-γ and 10 µM TNFα (HaCaT) for 24 h, and then supernatants were collected. The amount of cytokines released was determined using the BD OptEIA human IL-6 ELISA kit (BD Biosciences, Franklin Lakes, NJ, USA).

### 4.7. Western Blotting

The expression of Smad3 and pSmad3, as well as Smad2 and pSmad2, in cells was determined by Western blot analysis. Cells were incubated with syringin at a concentration of 100 µM for 24 h. TGFβ (10 ng/mL) was used as a positive control. After incubation, cells were collected and centrifuged at 200× *g* for 10 min at 4 °C. Cells were lysed in ice-cold RIPA buffer (Sigma-Aldrich Chemie, Munich, Germany) containing phosphatase and protease inhibitors (Sigma-Aldrich Chemie, Germany), and the resulting lysates were centrifuged at 9000× *g* for 15 min at 4 °C. The protein concentration was quantified by a standard colorimetric test (Thermo Scientific, Waltham, MA, USA), and 15 μL of lysate was resuspended in 5 μL 4 × Laemmli Buffer, centrifuged, vortexed briefly, boiled for 5 min at 95 °C, vortexed, and frozen as aliquots at −70 °C until analysis by 12% SDS-PAGE. Protein in the amount of 40 μg was transferred to nitrocellulose filters and immunoblotted with anti-Smad3 (1:20,000), anti-pSmad3 (1:1000), anti-Smad2 (1:1000), and anti-pSmad2 (1:2000) (ABclonal, Düsseldorf, Germany) and a rabbit anti-β-actin polyclonal antibody at a 1:5000 dilution (Fine Test, Wuhan, China). Peroxidase-conjugated AffiniPure goat anti-rabbit antibody was used as a secondary antibody at a dilution of 1:1000 (Thermo Scientific, Waltham, MA, USA). Finally, the blots were incubated with chemiluminescent substrate for the detection of HRP (Thermo Scientific, USA) for 5 min. Western blot analyses were quantified using ImageJ 1.54g software after densitometric scanning of the bands (Bio-Rad, Hercules, CA, USA).

### 4.8. Quantitative PCR

Total cellular RNA was isolated using a High Pure RNA Isolation Kit (Roche Molecular Systems, Pleasanton, CA, USA), following the protocol provided by the manufacturer. The RNA concentration and purity were determined using the Nanodrop One Spectrophotometer (Thermo Scientific, Waltham, MA, USA). Reverse transcription of RNA and cDNA synthesis were carried out using the RevertAid First Strand cDNA Synthesis Kit (Thermo Scientific, Waltham, MA, USA). Briefly, 300 ng of each total RNA sample was reverse-transcribed using oligo-dT primers and RevertAid M-MuLV reverse transcriptase, according to the manufacturer’s instructions. The reaction conditions were as follows: 42 °C for 60 min (reverse transcription) and 70 °C for 5 min (enzyme inactivation). The final cDNA concentration was 15 ng/µL (in total RNA equivalents) and was stored at −20 °C until further use. Prior to cDNA synthesis, DNase treatment to remove trace amounts of DNA was performed using the DNA-free DNA Removal Kit (Invitrogen, Carlsbad, CA, USA) according to the manufacturer’s instructions. No reverse transcriptase controls (NRT) were included to detect potential genomic DNA contamination in RNA samples. The following genes were analyzed: Tissue inhibitor of metalloproteinase 3 (TIMP3), Collagen type I α1 (COL1A1), and α-smooth muscle Actin (ACTA2). Glyceraldehyde-3-phosphate dehydrogenase (GAPDH) and β-Actin were used as controls. A list of the used primers is reported in the Supplementary Materials Table S1. All primers were obtained from Genomed (Warsaw, Poland). Quantitative PCR was performed using the SYBR Green JumpStart Taq ReadyMix (Sigma Aldrich, St. Louis, CA, USA). Each reaction (25 μL total volume) contained 12.5 μL 2x JumpStart Taq ReadyMix, 0.05 μL of each primer (100 μM), 1 μL of the cDNA template, and nuclease-free water to 25 μL. Reactions were run in 96-well plates sealed with optical adhesive film. The thermal cycling conditions were as follows: initial denaturation at 94 °C for 2 min, followed by 40 cycles of denaturation at 94 °C for 15 s, annealing, extension, and SYBR detection at 60 °C for 60 s. Melt curve analysis was performed from 65 °C to 95 °C (0.5 °C increment every 5 s) to verify amplification specificity. Amplification was performed in a Bio-Rad CFX96 Real-Time System (Bio-Rad Laboratories Inc., San Jose, CA, USA). The presented data represent averages of three technical replicates for each biological sample, performed at least two times. No-template controls (NTCs) and no reverse transcriptase control (NRT) were included in each plate to monitor potential contamination. Quantification cycle (Cq) values were determined using the Bio-Rad CFX Maestro 1.1 software (Bio-Rad Laboratories Inc., San Jose, CA, USA) with automatic threshold settings. For relative quantification, the comparative ΔΔCt method was used. Fold changes in gene expression were calculated using the formula 2^−ΔΔCt^. The results are presented as relative expression levels, normalized to the control group, which was set to a value of 1. The qPCR experiments were designed and reported in accordance with the MIQE (Minimum Information for Publication of Quantitative Real-Time PCR Experiments) guidelines to ensure experimental transparency and reliability of results.

**Gene****Accession Number****Forward Primer (5′-3′)****Reverse Primer (5′-3′)**Target genesTIMP3NM_000362TACCGAGGCTTCACCAAGATGCCATCTTGCCATCATAGACGCGACCOLA1NM_000088GATTCCCTGGACCTAAAGGTGCAGCCTCTCCATCTTTGCCAGCAACTA2NM_001613CTATGCCTCTGGACGCACAACTCAGATCCAGACGCATGATGGCAReference genesGAPDHNM_002046GTCTCCTCTGACTTCAACAGCGACCACCCTGTTGCTGTAGCCAAβ-ActinNM_001101CACCATTGGCAATGAGCGGTTCAGGTCTTTGCGGATGTCCACGT

### 4.9. Statistical Analysis

The results were expressed as the mean ± SD of at least 3 independent experiments. Statistical significance of differences between means was established by ANOVA, followed by Dunnett’s multiple comparisons test or the Tukey post hoc test. *P* values below 0.05 were considered statistically significant. All analyses were performed using Statistica v. 13.3.

## 5. Conclusions

In conclusion, our study demonstrated that syringin may accelerate wound healing through the stimulation of cell migration and reduction in inflammation at the site of injury, as well as by stimulating the expression of TGF-dependent genes important in the wound healing process. Further studies on more advanced in vitro and in vivo models would be needed to confirm the efficacy of syringin as an agent that can accelerate the healing of hard-to-heal wounds.

## Data Availability

Data are contained within the article and Supplementary Materials.

## Supplementary Materials

The following supporting information can be downloaded at: https://www.mdpi.com/article/10.3390/ijms26167827/s1.
