# Supplementary material for: Syringin (Sinapyl Alcohol 4-O-Glucoside) Improves the Wound Healing Capacity of Fibroblasts and Keratinocytes In Vitro
Source: Int J Mol Sci. 2025 Aug 13;26(16):7827. doi: 10.3390/ijms26167827 (PMC12386873; doi:10.3390/ijms26167827)
Supplement: Supplementary file 1 [file ijms-26-07827-s001.zip › ijms-3682433-supplementary.pdf]

Table S1 Primer sequences used for RT-qPCR

	Gene	Accession number	Forward Primer (5'-3')	Reverse Primer (5'-3')
Target genes	TIMP3	NM_000362	TACCGAGGCTTCACCAAGATGC	CATCTTGCCATCATAGACGCGAC
	COLA1	NM_000088	GATTCCCTGGACCTAAAGGTGC	AGCCTCTCCATCTTTGCCAGCA
	ACTA2	NM_001613	CTATGCCTCTGGACGCACAACT	CAGATCCAGACGCATGATGGCA
Reference genes	GAPDH	NM_002046	GTCTCCTCTGACTTCAACAGCG	ACCACCCTGTTGCTGTAGCCAA
	β-Actin	NM_001101	CACCATTGGCAATGAGCGGTTC	AGGTCTTTGCGGATGTCCACGT
